# Extracts and Gleanings

**Published:** 1872-10

**Authors:** 


### PART II.

### Abridged Extracts and Gleanings from our Exchanges

*MULTUM IN PARVO.*

---

Diet for Infants.—(Proceedings of the Michigan State Society—Detroit Review of Medicine, July, 1872):

Dr. Wilson read a paper upon "Bottled Babies," or the feeding of babies who were deprived of their mother's milk. He had found that a mixture of milk and lime water, in varying proportions to suit each particular case, was the best substitute for mother's milk. He insisted on giving it regularly every two hours during the day, and every three hours at night; upon having the child lie with its mother; upon the most scrupulous cleanliness of apparatus used, and upon changing cows when the milk of one disagreed with the baby. In the treatment of infantile diarrhœa he had obtained great benefit from the use of cold water injections after the evacuations.

Incontinence of Urine in Children.—Dr. Holms Coot says that he has relieved incontinence of urine in children, by the use of creasote, in every case in which he has tried it (some six in all) and when all other remedies had failed. The manner in which he administered the creasote is in the form of pill, one minim, with a sufficient quantity of bread-crumb.—*Chicago Medical Examiner.*

Chloral for Toothache.—Dr. Page, in the British Medical Journal, recommends choral hydrate as a local application in cases of toothache. A few grains of the solid hydrate introduced into the cavity of the tooth upon the point of a quill speedily dissolves there; and in the course of a few minutes, during which a not unpleasant warm sensation is experienced, the pain is either deadened, or more often effectually allayed. A second or third application may be resorted to if necessary.—*The Druggist's Circular and Chemical Gazette.*

Carbolic Acid in Dysmenorrhœa—Dr. Duncan reports a case of obstinate dysmenorrhœa cured by direct application of carbolic acid to the os uteri, using at the same time, general tonics, to build up the system.—*The Medical and Surgical Reporter.*

Expectorant Mixture.—A correspondent in Canada sends us the following recipe which he has found very effective in treatment of Coughs, Colds, Asthmetic difficulties, etc.:

| | |
|---|---|
| R.—Tinct. Benzoin Co. - - - | Cong. iss. |
| Tinct. Sanguinariæ, | |
| Tinct. Lobeliæ, | |
| Tinct. Opii Camph., | |
| Syr. Scillæ, - - - - | aa Oiii. |
| Ol. Helianth Ann. - - - | ozs. v. |
| Ol. Sassafras. - - - - - | ozs. ii. |
| Mellis Colat. Opt. - - - - | lbs. xii.—M. |

Dose, a dessert spoonful every 3 or 4 hours, or more frequently if the cough is hard and expectoration difficult.—*Jour. Materia Medica.*

Odontalgia — Systematic Treatment.—Dr. A. L. Cory, (Chicago Medical Times) "Reports a new remedy for toothache. Being called upon immediately after the fire, to extract some aching teeth, and not having the necessary implements, and most of the dentists having lost their instruments, he prescribed 20 grains of hydrate of chloral, to lessen the pain and procure rest until he could obtain the necessary forceps. To his astonishment, in every case, the ache not only promptly vanished, but failed to return. The Dr. says he has now tried this plan in about 20 cases with quite uniform success."

Various anodynes will answer the same purpose. Among others, iodoform in one grain doses is a very efficient remedy for dental and facial neuralgia.—*Ed. Medical Cosmos.*

Case of Keloid Treated by Iodide of Potassium.—Under the care of Dr. Davis, there is a man suffering from a considerable development of keloid in some old syphilitic scars, with which his body was pretty freely sprinkled. Under ten grain doses of the iodide of potassium, the keloid was rapidly disappearing.—*Lancet.*

Iodine in Vomiting.—Dr. Caspari, of Horn, has for the last 25 years successfully prescribed the tincture of iodine in cases of obstinate vomiting, where nothing else relieved the patient.

TREATMENT OF TRUE CROUP BY INHALATION OF GLYCERINE MYRRH AND CARBOLIC ACID.—Dr. N. M. Meek, (*Medical and Surgical Reporter*), remarks:

My brother, aged three years, was taken with true croup, having the peculiar cough, *croupal cough*, accompanied by sonorous or stridulous breathing. He had slight fever and flushed face for a week before the above symptoms set in; having no catarrhal symptoms whatever. The usual treatment employed in this disease was applied, but without any benefit. Having lost a case the week previous I determined to try some other treatment on this case. I ordered the following treatment.

R.—Glycerine,
Tinct. Myrrh, - - - aa. dr. ijss.
Carbolic Acid, - - - - grs. ij.—M.

To be applied every six hours, through the complete steam atomizer.

It was applied for the first twenty-four hours with but very little relief to the patient, when I ordered it to be applied every hour for five or ten minutes at a time. When the third application had been made the cough became more free and moist. The applications were made every hour for fifteen hours, when there was free secretions from the nose, with expectorations of mucus and purulent matter. Recovery from the disease is the result of the above treatment. I have used the same treatment in scarlatina anginosa and diphtheria with good success, reducing the inflammation and cooling the parts. The effect of the medicine is supposed to increase the secretions of the mucous membrane and reduce tumefaction. Whether there is anything in this as a treatment for true croup is to be determined by further trial. Hoping to hear from some one, I am yours, with respect,

IVY POISONING.—Mr. H. Markham, of Port Jefferson, New York, sends the following note to the *Scientific American:*

"I send you a prescription which, I am satisfied, from ten years' experience, is the very best remedy for ivy poisoning. It is simply to bathe the parts affected with *spirit of nitre.* If the blisters be broken, so as to allow the nitre to penetrate the cuticle, more than a single application is rarely necessary, and even where it is applied to the surface of the skin three or four times during the day, there is rarely a trace of the poison left the next morning."—*Druggist's Circular.—Chi. Med. Jour.*

THE MUMPS.—Dr. Eldridge says he had invariably cured mumps even when *orchitis* had taken place with an emetic in twelve, fourteen, or twenty-four hours. He never knew the emetic to fail.—*The Doctor.*

CHOREA.—Dr. Edward Montgomery (North-western Medical and Surgical Journal, July, 1872), speaking of the treatment of Chorea, remarks:

"When the disease depends on ænemia, mal-nutrition, or an altered condition of the blood, favorable hygienic measures with tonic medicaments are indicated. In these forms of the disease strychnine, quinine, arsenic, zinc, iron, etc., are the medicines from which most good is to be expected. The following is a favorite prescription of mine in these cases:

| | |
|---|---|
| R.—Strychnia, - - - | gr. i. |
| Quininæ Sulph. - - - - | dr. i. |
| Ascidi Phosphorici Dilut., - - | fl oz. i. |
| Aq. Menth, Virid, - - - | fl oz. vi. |
| Tinct. Cardum. Comp. - - | fl oz. i. Misce. |

Sig. A teaspoonful three times a day, for a child 8 or 9 years of age.

The citrate of iron and quinine, or Fowler's solution of arsenic with the tincture of cimicifuga racemosa, are remedies well adapted to this form of the disease.

When there is exalted nervous sensibility or hyperæsthæsia, musk, asafœtida, castor, valerian, zinc, etc., will be found of essential service. A very good prescription is the following:

| | |
|---|---|
| R.—Moschi, - - - | gr. xii. |
| Zinci Oxidi., - - - | gr. xxiv. |
| Sacchari, - - - - | dr. i.—Misce. |
| Divide in chart. No. xii. | |

S. One powder three times a day, for a child aged 8 or 9 years.

The only objection to the above is the expensiveness of the musk.

Another very excellent compound in these cases, is this:

| | |
|---|---|
| R.—Zinci Sulphatis, - - - | gr. xxx. |
| Ext. Valerian, (fluid) - - - | fl oz. i. |
| Syrup Limonis, - - - - | fl oz. iii.—Misce. |

Sig. A teapoonful three times a day, gradually increasing the dose.

I have found this prescription act most happily and promptly, stopping the choreic movements in a few days. To a watery solution of the sulphate of zinc may be added the tincture of castor, musk, cimicifuga; or any of these tinctures may be added to Fowler's solution, or the fluid extract of valerian, and it is well to have a variety of forms so as to administer that combination which is most grateful and most efficacious to the patient. If pills can be swallowed, zinc and asafœtida can be given in that form with very flattering prospects of success. We have very great faith in the efficacy of zinc in a great many cases of chorea; either the oxide or the sulphate will answer; we should gradually increase the dose and keep giving the medicine until the desired results are obtained. We have such confidence in the efficacy of zinc in the great majority of these cases, and we have had such remarkable success with it that we consider it almost a specific; a great many years ago we cured a young woman aged twenty-three, who had been constantly suffering with the disease for ten years; in this case, besides the zinc, we prescribed the strychnine and quinine mixture; and also gave Vallet's carbonate of iron pills freely. It is important to pay attention to the bowels; when sluggish or torpid the following prescription will be found particularly appropriate in this, as in many other diseases attacking patients of weakly or nervous constitutions.

| | |
|---|---|
| R.—Ext. Belladonnæ, - - - | gr. ii. |
| Ext. Nucis Vomicæ, - - - | gr. iv. |
| Ferri Sulph., Exsicat - - | dr. ss. |
| Pulv. Aloes Soc., - - - - | scr. ii.—Mix |

And divide into twenty pills, and give one night and morning, or one every night as the case may require, until the bowels become perfectly regular.

We can confidently recommend the above pills in cases of chronic constipation; by taking one every night and morning for a short time, then one pill a day for a few days, then one pill every two or three days, in a few weeks the medicine can be dispensed with entirely. If the patient cannot swallow pills, the following may be substituted for them:

| | |
|---|---|
| R.—Ferri Citratis, - - - - | dr. i. |
| Tinct. Belladonnæ, - - - - | fl dr. i. |
| Tinct. Nucis Vomicæ, - - - | fl dr. ii. |
| Tinct. Aloes, - - - - - | fl oz. ii. |
| Syrupi Limonis, - - - - | fl dr. xiii.—Mix. |

And give a tea-spoonful, or more, according to age, every day, or more frequently if necessary. This formula, for an aperient mixture we have found a very excellent one; it does not nauseate or sicken the patient, but on the contrary gives tone and energy to the digestive organs and removes the tendency to returning constipation, thereby obviating the necessity of giving frequent purgatives.

If we suspect endocardial disease, embolism, arachnoid effusion, or softening of the nervous centers, we may not be able to accomplish much with medicines. but even in these cases we would urge the employment of the iodide or bromide of potassium, carbonate or hydrochlorate of ammonia, etc. The most favorable hygienic conditions are necessary in these cases.

VOMITING OF PREGNANCY.—Dr. De Camp read a paper upon the treatment of the vomiting of pregnancy. He shewed that oxalate of cerium failed in one-half the cases. Arsenic, he regards as the most efficient of all remedies. His attention was first called to the use of the remedy in 1858 by observations upon its effects in cases of poisoning. In his experience nine-tenths of the cases were cured by arsenic. He used it in doses of one-thirtieth of a grain, or Fowler's solution in like proportion. Calomel, he found of value in those cases in which bile was thrown up with the vomiting—dose, one-tenth of a grain. Though he had used other remedies, he had come to depend entirely upon the three already mentioned, and had no fear of controlling the disagreeable symptoms. In cases attended with acidity, he gave oxalate of cerium; those with loathing of food, arsenic; those with vomiting of bile or excess of salivary secretion, calomel.—*Ibid.*

TAPE WORM.—Dr. S. R. Knight, in the Medical Times, recommends the following:

| | |
|---|---|
| Kameela, - - - - | oz. ss. |
| Simple Syrup, - - - - | f oz. j.—M. |

*Dose.*—A tablespoonful. One dose will generally be sufficient to expel the worm.

TREATMENT OF ENUROSIS.—Sponging the lower part of the spine before going to bed is said to have greatly benefitted this troublesome complaint in children, when all other treatment failed.

Dr. J. H. Hollister relates a case of chronic conjunctivitis, which was rapidly cured by the application of an ointment of the biniodide of mercury to the outside of the eye-lids.

Electrolysis in Ovarian Tumors.—Dr. F. Fieber reports (Wien Med. Presse) the following: "A seamstress, aged 32 years, had a tumor of the size of a man's head, slightly painful on pressure, hard, nodulated, extending from half an inch below the umbilicus down to within an inch above the symphysis; its breadth was six inches. Diagnosis: ovarian cyst of the right side.

"June 6th, 1870, a large gold needle with a copper pole, connecting with Daniel's battery of twenty elements, was inserted one and a half inches deep into the swelling, one and a half inches below and to the right of the umbilicus, the chain was then closed to the left of the umbilicus. While the needle was inserted, the patient experienced a sensation of severe burning, which, however, soon disappeared; after seven minutes the needle was withdrawn: reaction slight.

"Up to November 30th, electrolysis was repeated eleven times, subsequent inflammatory symptoms but rarely requiring poultices and small doses of morphine. The needle was inserted into different places. Till the end of November, the swelling diminished but little; afterward it decreased rapidly. March 10th, 1871, the cyst was reduced to the size of a hen's egg.

Dr. F. urgently recommends in every case the employment of electrolysis before undertaking ovariotomy.—*Medical Archives.*

In cases of fever, with (or without) marked gastric irritability, Prof. Dickson gave the following:

| | |
|---|---|
| R.—Potassæ bi carb., . . . | scru j. |
| Tinct. opii camphor, . . | f dr ij |
| Aquæ Puræ, . . . | f oz viij.—M. |

S.—A tablespoonful every two hours.

Eucalyptus.—Dr. David Wooster, in an article printed in the Pacific Medical and Surgical Journal, says of the fluid extract of eucalyptus:

"I have now used some gallons of this extract during a period of eight months in the U. S. Marine Hospital, and am surprised at its uniform and reliable effects in affections for which it is suitable. It is a diuretic of rare virtue, and may be administered when most of the diuretics now in common use are inadmissable. It is an aromatic tonic, and has notable restorative effects in low states of the system, as in typhoid fever, typhoid diarrhœa and dysentery.

"In vesical catarrh it alone cures. In spasmodic stricture it relieves with great promptness. In all affections of the mucous membranes its beneficial action is remarkable. We have treated many cases of acute gonorrhœa with no other remedy. Per contra, in syphilis it is useless except as an alterative.

"It has no anti-periodic action. It relieves headache apparently as the saline diuretics do, by its diuretic action.

"As an external application in chronic ulcers, it has great value.

"It does not impair, but rather improves, the appetite.

"It is to be hoped the profession will try it more generally. I have now used it in some hundreds of cases. Dr. Coleman, formerly of Stockton, now in this city, first called my attention to its virtues, and I have used the fluid extract of his manufacture. Indeed, I believe it has not yet been manufactured by any other party."

For Enlarged Glands.—

R.—Iodide of lead, . . . dr i.
Soft cerate or lard, . . . oz j.
Mix exactly.

Apply frequently to enlarged lymphatic glands. Useful in inflammatory diseases of scrotum.

Calabar Bean in Cerebro-Spinal Meningitis.—Dr. Henry G. Todd, (Indiana Jour. of Med.), says:

In two recent cases in which I have used the bean, however, I have been able to give personal attention, pushing the remedy more vigorously, and with results equal to my most sanguine expectations. In both of these cases, (which were adults), I used the pulverized bean, as giving a better opportunity to graduate the dose. I commenced with four grains of the powdered bean, and repeated with three-grain doses every hour till relief was obtained from the urgent distress occasioned by the tension of the muscles of the neck and back. This object was partially attained after the third dose, but comparative ease was secured after the fifth, and the remedy was continued at such intervals as would give the patient comparative rest and comfort; but in neither of those cases was it pushed far enough to produce contraction of the pupil, though they complained of the depression. This treatment was continued in one of the cases about ten days, the other a shorter time, when the disease appeared so far recovered that the bromide of ammonia was substituted for the bean, and my patients recovered.

REMOVAL OF CORNS.—Hard corns may be carefully picked out by the aid of a small sharp-pointed scalpel or tenotomy knife, and if well done the cure is often radical, always perfect for the time. But they may be equally successfully removed by wearing over them for a few days a small plaster made by melting a piece of stick diachylon (emplastrum plumbi), and dropping it on a piece of white silk. The corn gradually loosens from the subjacent healthy skin, and can be readily pulled or picked out. Soft corns require the use of astringents, such as alum dissolved in white of egg, or the careful application of tincture of iodine. Prevention, however, is in regard to them better than cure, and can be readily attained by daily friction with cold water between the toes.—*Edin. Med. Journ.*, June, 1872.

FOR RHEUMATISM AND ARTHRITIS OF SYPHILITIC PATIENTS.—
R.—Guaicum wood,
Sarsaparilla root,
Peruvian bark, . . . aa oz ss.
Boiling water, . . . o j.
Macerate 24 hours, and then boil down to oz. vj—strain and express. Acts as a powerful diurectic and diaphoretic; causes a sensation of burning in the throat, which however, is of short duration.

HYDROTHERPY IN ECLAMPSIA.—In the Berlin Obstetrical Society Jacquet recommends in the treatment of eclampsia the priesnits envelopments of the whole body. The method is as follows: a woolen wrapper, 3½ yards long and three yards wide, is spread out over the bed. Upon this is laid a linen cloth of the same size which had been soaked in cold water, 18° R., and afterwards wrung out. The patient is now to be enveloped in this cloth, leaving free the head, which is covered with an ice cap. If pains have already commenced the legs must be separately enveloped in order that the wrappings remain in contact. In the course of an hour an abundant secretion of sweat takes place to last for several hours and gradually disappear. Under this influence the attacks diminish in number and frequency, and the patient falls into peaceful sleep without interruption of the course of labor. The author bases this recommendation, theoretically upon both views—the Frerich—Leitzman and Traube—Ronsenstein—of the nature of the affection and practically upon the results of its adoption in clinical experience. Eight cases treated in this way in the Royal Obstetrical Institution were rapidly cured. In the discussion which followed, Prof. Martin spoke of this means as one highly to be recommend, particularly in conjunction with treatment by chloral in clysmata, by chloroform, opium and morphia.

SYPHILITIC GUMMATTA OF THE LIDS.—Galezowski reports two cases of this form of syphilis. These tumors are not simple hyperplasia, but approach more nearly the heteroplasia. Those the author met with were situated deep under the skin ; very probably developed in the fibres of the orbicular muscles.

In one case the tumor was situated about the middle of the lower lid, in the other over the region of the lachrymal sac, simulating a lachrymal tumor.

These gummata were not painful, the skin was neither red nor inflamed.

Treatment.—Iodide of potass., large doses, and paint the tumor with tincture of iodine.—*Journal d'Ophthalmologie,* May, 1872.

FOR MERCURIAL SOMATITIS.—

| | | |
|---|---|---|
| R.—Tinc. of iodine, | . . . | dr ii. |
| Tinc. of nutgalls | . . . | dr ii. |
| Water, | . . . . | o j.—M. |

The addition of an ounce of the tincture of orange-peel, or of cinnamon water will modify the unpleasant taste.

AMYLOID DISEASE PREVENTED BY ALKALIES.—James Perrigo, M. D., London, correspondent of the Canada Medical Journal, writes that Dr. Dickinson, of the Great Ormond street Children's Hospital, is a great believer in the theory that alkalies can prevent amyloid disease in long-continued suppuration. This is in the face of opinions recently held, of alkalies long administered causing anæmia by lessening the number of red corpuscles. A case was shown on the surgical side, suffering from a suppurating hip-joint, where the patient had been taking citrate of potash for eighteen months, and no amyloid disease was present.

MOUTH WASH.—

| | | |
|---|---|---|
| R.—Tinct. of nutgalls, | . . | dr ii.—oz ss. |
| Water, | . . . . . | o j. |

Of special service in the treatment of flabby soft gums which bleed easily. An ounce of tincture of orange-peel will improve the taste.

MANAGEMENT OF PERSISTENT HICCOUGH.—Dr. Emory L. Willard (Pacific Medical and Surgical Journal), relates three cases of violent and persistent hiccough, which were relieved by inhalation of the salts of white hellebore. The hiccough stopped as soon as the new action was set up by the remedy.

PHENOL SODIQUE.—This preparation of carbolic acid is deservedly popular with the medical and dental professions. Dr. E. Wildman gives the following formula in the *Dental Times* for its preparation:

| | | |
|---|---|---|
| R.—Carbolic acid, in crystals, | . . | 188 gr. |
| Caustic soda, | . . . . | 31 gr. |
| Pure water, | . . . . | fl oz 4.—M. |

The carbolic acid should be free from offensive odor, such as is prepared for medicinal purposes. When first mixed, it is nearly colorless, but in time assumes a wine color; does not deposit any tarry residue, to often found in the commercial article. This formula is the result of numerous experiments, and gives an article that will compare favorably with the best French Phenol Sodique.

A ZOOTROPHIC POWDER.—V. Polli, after dwelling on the importance of the various mineral constituents of the body in food, if the healthy action of the tissues is to be maintained, recommends the following powder as containing some of the most essential: Phosphate of lime, 10 parts; tribasic phosphate of lime, 10 parts; phosphate of soda, 15 parts; carbonate of lime 10 parts; sulphate of magnesia, 15 parts; chloride of sodium, 10 parts; bicarbonate of potassa, 15 parts; oxide of iron 10 parts; oxide maganese, 2.5 parts; silicate of potassa, 2.5 parts. The dose of this powder is 45 grains daily for children, or double this quantity for an adult. He recommends its use especially—1. In infants at the breast, who are suffering from dentition, or in nurses. 2. In children suffering from osteomalachia, rachitis, scrofulosis, or chlorosis. 3. In pregnant women, and those suffering from puerperal cachexia. 4. In fractures, and cases of caries of the bones. 5. In tuberculosis, especially when there are vomicæ. 6. In anæmia after hemorrhages, and in leucocythæmia. 7. In convalescence after long illness.—*Medizinish Chirurg.*, *Rund.*, *from The Practitioner.*

PEPSINE IN DIARRHŒA.—Dr. Davidson, of the Children's Infirmary, of Liverpool, Eng., calls attention, through the Practitioner, to the prompt benefit which follows the use of pepsine in certain forms of diarrhœa occurring in young children. There is debility of the digestive organs; unassimilated food passes away by frequent motions, and there is often action of the bowels immediately after eating, the ingestion of food into the stomach apparently exciting the movement of the bowels. The mother states that food passes through the child as soon as it is eaten. Astringents and antacids do no good; but the writer has found that small doses of pepsine, taken three or four times a day with the food, scarcely ever fails to restore health..

Syrup of Coffee.—This preparation is of great use to those who have long journeys to make. Take ½ lb. of the best ground coffee; put it into a saucepan, containing three pints of water, and boil it down to one pint. Cool the liquor, put it into another saucepan, well scoured, and boil again. As it boils, add white sugar enough to give it the consistency of syrup. Take it from the fire, and when it is cold put it into a bottle, and seal. When traveling, if you wish for a cup of good coffee, you have only to put two teaspoonsful of the syrup into an ordinary coffee-pot, and fill with boiling water. Add milk to taste, if you can get it.—*Food Journal.*

Bone Felon Arrested.—Dr. James B. Walker details, in the Medical Archives for March, 1872, a case of bone felon cured in an early stage by the finger being put into a mixture of ice and salt until well congealed, then taken out, and, when the pain came back, replaced, and so on for two hours. On the first application of the cold there was intense pain for a little while.

Emetia.—Dr. Duckworth stated to the Pharmaceutical Association of London, that friends of his had used emetia largely in the treatment of dysentery in India, and had obtained from it all the good effects of ipecacuanha. The dose employed was from the sixth to a twelfth of a grain, with or without morphia, as the indications seemed to require.—*Pharmaceutical Journal, March* 9, 1872.

Calabar Bean in Chorea.—Dr. T. P. Russel says of calabar been (Northwestern Surgical and Medical Journal): I have used it with great satisfaction in long-standing cases of Chorea. In one case, of more than one year's standing, a perfect cure was obtained.

Chloral.—Dr. Leibreich, in the third edition of his treatise on chloral, calls attention to the fact that, if the drug produces excitement in a patient, the same dose will have a hyponotic effect after treatment with carbonate of soda. When the blood is alkaline the chloral is rapidly changed into chloroform.—*British Medical Journal.*

Local Anæsthesia in the Use of Caustics.—M. D. H. Spessa asserts (L'Imparziale) that if a hypodermic injection of morphia be given in the tissue to be acted on just before the application of the caustic, the latter will cause little or no pain.—*Journal de Pharm. de Chimie.*

CALABAR BEAN IN EPILEPSY.—Dr. S. D. Williams has been using calabar bean in epilepsy. About half of the cases (12 in all) were apparently benefited by the drug, the others not. It did not interfere perceptibly with nutrition, although the effect on the pulse and temperature was marked, the pulse being reduced from five to ten beats per minute, and the temperature from a half to two degrees. In many cases there was marked indolence and decided flaccidity of the muscular system. Increased action of the cutaneous glands was quite decided.

ILEUS CURED BY ELECTRICITY.—Dr. Michael Bogden, in the Wiener Medicinische Presse of March 10th, 1872, details a case of ileus cured by electricity. He introduced one pole of an induction apparatus into the rectum, as far as he was able, and then placed the other over the spot where an intussusception was believed to exist, and passed at first a primary, afterwards a very strong secondary current, which produced very great distress and vomiting. Shortly afterwards a large quantity of wind and four ounces of clear odorless fluid were passed. After this there were no more severe pain or vomiting. During the succeeding night very profuse diarrhœa came on, which was finally checked by Dover's powder. Under the exclusive use of a milk diet, the patient speedily convalesced.

DRIED BEEF PULP.—G. Dannecey states that beef pulp, spread upon muslin and exposed to a current of warm, dry air, soon becomes converted into a brown mass, without odor, which represents five to six times its weight of raw beef; is readily eaten in sandwiches, or even diffused through soup, and possesses all the therapeutic value of raw meat.—*L'Union Pharmaceutique, March,* 1872.

STRYCHNIA IN VOMITING.—In the vomiting of hysteria, pregnancy, ovarian tumors, suppressed menses, and uterine disease, M. Debauge, in the Lyon Medical for January, 1872, commends strychnia given by the mouth and hypodermically.

TETANUS.—In the Pacific Medical and Surgical Journal, March 1, 1872, Dr. Clinton Cushing reports a case of tetanus, in which he thinks the successful issue was largely due to the use of morphia and chloral in combination.

THE Oxalate of Potash in the treatment of peritonitis is recommended in the Jour. de Med. et de Chir., small doses in mucilage, repeated every hour, having produced good results in three cases of puerperal origin.—*Med. Record.*

A Case of Calculus Nephritis.—Dr. Wm. Chambers, of Effingham, Illinois (Chicago Medical Examiner), reported a case of calculus nephritis in which recovery followed the subjoined treatment:

Externally, a blister; internally, with the following mixture:

| | | |
|---|---|---|
| R.—Potass. acetatis | - - - - - | oz. ss. |
| Vini colchici | - - - - - | oz. ss. |
| Spts. nitrosi dulcis | - - - - | oz. ii. |
| Tinct. opii. camph | - - - - - | oz. ss. |
| Fl. ex. belladonnæ | - - - - - | dr. i. |
| Aq. cinnamoni | - - - - - | oz. ss.—M. |

Sig. One teaspoonful every three hours.

A calculus of phosphate of lime, weighing five grains, passed the urethra the day following.

Glycerole of Tannin.—R. Rother, in the Chicago Pharmaceutist, commends the following formula:

| | | |
|---|---|---|
| Take of Tannin | - - - - | 8 troy ounces. |
| Glycerine | - - - - - | 4 " " |
| Strong alcohol | - - • | 8 fluid " |
| Water | - - - - | 8 " " |

Mix the alcohol and water; add the tannin, and apply heat until the tannin has dissolved. Filter hot, then add the glycerine and evaporate by a careful heat until the solution weighs 16 troy ounces.

Treatment of Diabetes Insipidus.—M. Gueneau de Mussy, in a clinical lecture at the Hotel Dieu, recommends the administration of full doses of belladonna, and sulphurous baths, in the treatment of diabetes insipidus. He has twice found belladonna to accidently produce anuria. Its use in incontinence of urine is well established. Systematically employed in diabetes insipidus, it has diminished the quantity of urine passed from ten pints to two pints *per diem.* The sulphurous baths brings the skin to the relief of the kidney.—*Brit. Med. Journal.*

Coryza.—Dr. C. Barbier gives the following advice with regard to the treatment of this affection:

1st. Avoid sneezing, and blow the nose as little as possible, since these are sources of irritation to the Schneideran membrane.

2d. Use the following powder as a snuff, three or four times a day, drawn in strongly until an effect is produced: Nitrate of Silver, 1 part; Sugar, 9 parts. Reduce to an impalpable powder.—*Lyon Medicate.*

Distilled Lavender Water is preferred by Dr. Delioux, and regarded as superior to rose and plantain water in affections of the eye; in slight opthalmias it answers as a wash, for severer ones it is the best vehicle of collyria.—Rep. de Pharm., 1872, June, 458. Bullet. therap.—*Am. Jour. Pharmacy.*

Koussin in Tapeworm.—In writing of this remedy Dr. C. Bedall, Munich, (Am. Journal Pharmacy), arrives at the following conclusions:

1. Koussin is the only active principle of kousso, and deserves the preference before the latter.
2. It is preferable to other tænifuges, because 2 scruples=2.5 grm. are sufficient for dislodging the tapeworm, and the remedy, divided according to age and constitution into two or four powders, is conveniently taken between wafers, and usually agrees well with the patient, producing in exceptional cases, merely transient nausea or vomiting.
3. In the doses mentioned, koussin leaves no ill effects of any duration; on the contrary, most patients enjoy good health and appetite after the tapeworm has been expelled.
4. Koussin needs no preparatory treatment in diet or with other remedies; but in obstinate cases it may be advisable to aid its action by giving some epsom salt or other convenient purgative.
5. If, after the use of koussin, the tapeworm should not be entirely expelled and its small head not be found, it is well to ascertain whether it has not been killed and the head is not subsequently discharged.
6. If the treatment be unsuccessful, this should not be charged to the koussin, but rather to casual circumstances which counteract, more or less, the effects of this remedy.

Bullock's Blood—A New Remedy.—M. Boussingault says: In the practice of medicine, as in other worldly matters, certain things are in fashion for a certain time. Bleeding and mercury have had their day; cod-liver oil and chloral hydrate are already on the wane; alcohol and bullock's blood are now in vogue among the Parisians—the former for fevers and all inflammatory affections, and the latter for anæmia and pulmonary phthisis. It is a curious sight to see the number of patients of both sexes and of all ranks and ages who flock to the slaughter-house every morning to drink of the still fuming blood of the oxen slaughtered for the table. I was struck at the facility with which young ladies take to it, and I have heard many say that they prefer it to codliver oil. I shall not enter into any theoretical speculations as to its *modus operandi*, but what I can vouch for is, I know of several cases of anæmia that have been cured and some of phthisis pulmonalis greatly benefitted by the treatment, at least, as much as they would be under cod-liver oil. For the more fastidious, however, a pharmacien has prepared an extract of blood, which is administered in the form of pills, each of which, weighing about three grains, is said to be equivalent to about half an ounce of pure blood.

A distinguished chemist lately read a paper before the Academy of Sciences, giving an account of his researches on the composition of the blood, and expressed his surprise that, containing as it does all the constituents of a perfect aliment, it is not more generally employed as a food. This is a subject worthy the consideration of philanthropists, especially in these days, when the price of meat is everywhere steadily increasing—at least, among the meat-eating population; and it strikes me that the rivers of blood that are daily spilt on the ground in slaughter-houses might be utilized as food. In Europe, pig's blood is the most generally consumed in the form of sausages; but that of all animals, without distinction, might in this way be more usefully employed. It is well known that in the steppes of South America the natives have for a long time used as food the blood of the animals they chased, which they previously coagulate and season with different condiments.

USE OF THE ESSENCE OF EUCALYPTUS GLOBULUS TO DISGUISE THE ODOR AND TASTE OF COD-LIVER OIL.—The researches of Prof. Gubler on the *Eucalyptus Globulus* and its essence—Eucalyptol—has suggested to M. H. Duquesnal a trial of the effect of the Eucalyptol in masking the disagreeable flavor and odor of cod-liver oil, and the result, he says, has been most satisfactory. He mixes one hundred parts of cod-liver oil with one part of the essence of eucalyptus. The oil thus aromatized, he states, has neither the taste nor odor of cod-liver oil; it is readily swallowed and leaves in the mouth or on the tongue only the flavor of the essence in which it is mixed; and the disagreeable eructations which follow the taking of the pure oil are completely modified. The aromatic oil may be kept for a long time if the bottle in which it is placed be maintained very closely stoppered.—*Med. News and Library, August*, 1872, *from Rev. de Therap., June* 15, 1872, *from Bull. de Therap.*

A STYPTIC "CURL PAPER."—Dr. James Thompson, (London Lancet), says:

I desire to introduce to the notice of the profession a styptic paper which I have found useful, and which has been successfully used by several friends in this neighborhood. It is prepared by Messrs. Cutting & Son, of Leamington, from directions furnished by me. Sheets of household paper, of moderate thickness, are steeped in a solution of tannin, of such strength that each sheet is impregnated with two grains, and then dried in the air. I have found them most useful in cases of external hæmerrhoids, used as closet paper, a portion being placed in the cleft of the anus after each use; also in excoriations around the anus caused by the acid discharge of diarrhœa in children. They form a ready styptic for stopping the bleeding from leech-bites, and in superficial excoriations from blows or falls. Many other modes of application may occur to your readers.

Injection of Ammonia as an Antidote to Chloroform Poison.—The efficacy of this method has been tried recently in Melbourne, with very encouraging results. A person, while under the influence of liquor, drank an ounce of chloroform in two doses, and became insensible almost immediately. An emetic of salt and water, which was poured down his throat without delay, caused him to vomit. The injection of liquor ammonia into the veins was decided upon. Half a drachm of liquor ammonia was injected into a vein in the arm, the strength of the injection being one part of ammonia to two parts of water. The good effect upon the pulse was rapid and marked, and, after an interval of twenty-five minutes, another half-drachm was injected. The improvement increasing, a third injection was made, after another interval of twenty-five minutes. After three-quarters of an hour, another half-drachm of ammonia was injected, bringing the total amount of *antidote* introduced into the blood to two drachms. The patient regained consciousness about midnight, and, with the sanction of his medical attendant, was removed, between seven and eight next morning, from a house of ill repute, where he had been staying, to his own residence. The deceased subsequently complained of weakness, and expired twenty-four hours afterwards.—*Medical Press and Circular.*

Cure for Intemperance.—It is stated that the use of the water of the Greenbrier White Sulphur Springs, Virginia, has been the means of curing drunkards.

Chalybeates in goitre are affirmed by Dr. Seitz to be very injurious. He believes that iron often causes the disease. The iodide of potassium should be administered to such patients.—*Cincinnati Lancet.*

XYLOL.—Dr. Richard Moffett, of Philadelphia, Pa., (Medical Times, June 15, 1872), says ;

This is a new remedy, recently discovered by a German chemist, and is used at the Royal Hospital in Berlin, in the treatment of small-pox. It is found in wood-tar and coal-gas naphtha. My first experience in the use of the remedy was in the following case. I was called to Mrs. Sophia H., a German woman, aged forty-two years, on March 27. She was suffering with preliminary symptoms of small-pox, which in a few days developed into the confluent form. My usual treatment failed to give any relief whatever, and she was fast sinking. On April 8 her pulse was 155, respiration 40, tongue brown, dry and hard ; the ends of her fingers and nails were purple, and her face was entirely covered with black scabs. The tonsils and parotid glands became so much affected that it was with the greatest difficulty that anything could be swallowed. She suffered from great restlessness, and was unable to obtain sleep even after taking large doses of chloral and morphia. Having obtained some of the new remedy —xylol—I determined to try it in her case. I gave her the following prescription:

R.—Olei xylol., . . . gtt cc.
Pulv. acaciæ, . . . q s.
Syrupi simplic,
Aquæ, . . . . aa f oz j.
S.—A teaspoonful every two hours.

I called the next day, and found her sitting up in bed. All the graver symptoms had disappeared. Her pulse was quite moist, pulse 98, respiration 22. She told me the medicine relieved her at once ; and her husband said that after taking three doses she went to sleep and slept for four hours.

April 14.—The patient is quite talkative, and can swallow without difficulty. From this time forward her convalescence was uninterrupted. At this date, April 19, she is able to go about the house, suffering only from a partial loss of the right eye. She was vaccinated when an infant, but bore no mark. I have tried this remedy in a number of cases since, and its use has always been attended with the most happy results.

COCOANUT MILK.—The London Pharmaceutical Journal states, that in India the milk of the cocoanut is employed in debility and incipient phthisis as a substitute for cod-liver oil, with excellent results. It is also used instead of cow's milk in tea and coffee. In large doses it acts as a substitute for castor oil.

EPILEPTIFORM TIC DOULOUREUX.—Dr. Roberts Bartholow (Clinic, June 1st, '72), remarks :

Cases of epileptiform tic douloureux are justly regarded as the most horrible aspects of suffering, and the most intractable as regards cure, of any with which we have to deal. The terrible operation of excision of the trunk of the fifth nerve near the Gasserian ganglion, eagerly borne by patients to be relieved for a time of their atrocious sufferings, is, as we all know, of temporary benefit, the neuralgia returning after some months with all its original intensity. Hitherto cases of this kind have not been cured by remedial agents, although the hypodermic injection of morphia and the galvanic current have afforded a temporary assuagement. By the persistent use of the hypodermic injection of morphia extending over a period of two years, and the conjoined use of the iodide and bromide of potassium, I have been enabled, fortunately, to cure a case of great severity which had resisted the efforts of some of our most skillful therapeutists.

Pain was the chief symptom. She described her sufferings as agonizing. Points sensitive in a high degree were found in various parts of the scalp. Wherever the pains had existed for any length of time, extreme tenderness remained. A nodular swelling exceedingly painful to pressure, had formed on the upper left side of the forehead, extending backward. Other nodes were formed at various times, and they were always extremely painful, but were fugitive—appearing and disappearing—whilst the larger one on the left side of the cranium continued until near the close of the treatment. The pains were of two kinds : superficial, corresponding in distribution to the ramifications of the fifth pair in the face and scalp, and deep as if located in the very centre of the brain. This deep pain was heavy, tensive, and accompanied with a sense of reverberation through the cranial vault. The pains were nearly continuous. The early hours of the morning were freest from them ; they came on about ten in the morning, gradually increasing in intensity towards evening ; deep pains in the brain, acute lancinating pains and soreness of the scalp. The act of eating and swallowing, bodily movements, a current of air, a gentle tap on the head, the rolling of carriages and wagons in the street, increased them.

It is not surprising that some evidences of impaired mental power were present. She talked slowly with a drawl, and had great difficulty in giving utterance to simple ideas. After her convulsive seizures she had hallucinations and illusions, religious frenzy, and uncontrollable excitement of a melancholic character. These attacks gradually declined, and her usual mental state was restored in some days after the convulsive seizures.

She complained of an extreme degree of vertigo. When lying in bed she had a strong sense of floating off, or swimming in the air; when walking, especially in descending steps, she had difficulty in maintaining the vertical position, and it was necessary to afford support to prevent her falling. Various motor disturbances occurred from time to time. The muscles of the face supplied by the seventh nerve were frequently agitated by spasm, and she had similar attacks in the trapezius. In the convulsive paroxysms the head was drawn to the right.

From this brief description of the symptoms I pass to the subject of treatment.

As pain was the chief symptom, I advised the hypodermic injection of morphia. In a few days I raised the amount injected each day to two grains of morphia and 1-96 of atropia. Her daughter was instructed in the mode of using the hypodermic syringe, and the injections were continued for two and a half years. The good effects were these:

She was enabled to take more food.

She could retain the necessary medicines.

She was relieved of suffering.

She was directed to take a pint of cream daily in addition to her other food, and she took dr. i of the syrup of the hypophosphites three times a day. As she was much emaciated, and as waste was going on rapidly, it was considered desirable to supply these important foods to the organism. Subsequently she was directed a half-drachm each of the iodide and bromide of potassium twice a day, and this was kept up steadily for several months. This medicine was administered after she had come under the influence of the hypodermic injection, which enabled her to retain it without difficulty.

I may sum up in a few words the results of the treatment:

The neuralgia ceased.

The nodes disappeared.

The menstrual function was reestablished in perfect regularity.

She rose in weight from 82 to 149 pounds.

Her mind was restored. The function of hearing was not improved, and deafness remains the only sequel of the formidable lesions which must have existed.

The hypodermic injections were continued for two and a half years. A severe struggle was anticipated in the effort to discontinue them, but by very gradually diminishing the amount daily injected, they were, after six months of careful adjustment of the quantity entirely stopped without any jar to her nervous system. Three months have now passed since they were discontinued, and no difficulty or embarrassment of any kind has been experienced.

Enlargement of Spleen Treated with Hyposulphite of Soda.—Dr. Thomas Hill, of Danville, Mo., writes to the Richmond and Louisville *Medical Journal:*

It having been my fortune monthly to have to treat several cases of enlargement of the spleen, and finding all the methods recommended in the books unsatisfactory, I concluded to try the effect of hyposulphate of soda, and met with such good results that I am induced to send you a report of a few cases for the Journal.

Case I.—K. B., æt. eight months; male. This little child had been having ague and fever almost from his birth; his mother having suffered with them almost through her whole pregnancy. The whole family having ague and fever; living on a low mill-pond, I recommended a removal to a more healthy locality, and put them on quinine and iron; they all soon improved, except the baby. Upon carefully examining it, I detected the presence of a large ague cake, and made the following prescription:

| | |
|---|---|
| R.—Quinine, - - - - - | gr. viij. |
| Hyposulph. Soda, - - - - | gr. xvj. |
| Water, - - - - - - | oz. j. |
| Elix. Vitriol, - - - - - | gtts. vj.—M. |

Sig.—Give a teaspoonful every two hours. A poultice of hoarhound to be applied to the spleen. In two days the enlargement had subsided, and by the use of small doses of iodide of iron the child was soon restored to health.

Case II.—Was an adult male. In this case ten grain doses of hyposulphite of soda, with one grain of quinine every four hours, completed the cure.

An infant seven months of age. Spleen enormously enlarged, with a yellow-jaundiced appearance of skin; chills and fever every other day. I put this child under the same treatment as the first, with an occasional dose of hydrag. cum. creta., grs. ij., and in four days it was entirely restored.

I would here state, that in the treatment of ague and fever I have repeatedly used the hyposulphite soda, and generally with good results; but for it to act beneficially, I have found that it must be dissolved in plenty of water, or two ounces of water to ten grains for an adult.

I have treated a good many other cases, but think these examples enough to draw the attention of the profession to the remedy.

The mortality in London during the last week of June was at the rate of 22 deaths annually in every 1,000 of the population.

Blisters in Pneumonia.—Dr. C. J. B. Williams, the distinguished English authority, makes use of the following language in speaking of the treatment of pneumonia:

"My experience has taught me to put great faith in large blisters, both in asthenic pneumonia and in bronchitis, and I am confident that I have seen many lives saved by their means. Instead of being lowering, they give a salutary excitement to the circulation, and the copious serous discharge which they produce from the skin tends to relieve the congested lung without wasting the red blood that is so needed to sustain the functions. Small blisters tease as much as large ones, and are far inferior in the relief they afford."

Balsamic Cigarettes for Asthma, Etc.—Soak strong unsized paper in a solution of saltpetre; this dry, and treat first with tincture of cascarilla, and afterwards, when nearly dry, with compound tincture of benzoin; cut into squares of a suitable size, and roll into the form of cigarettes.—*London Chemist and Druggist's Compendium.*

Compound Arsenical Paper.—

| | | |
|---|---|---|
| Take of | Belladonna Leaves, - - | grs. xcvj. |
| | Hyoscyamus " | |
| | Stramonium " - - | aa. grs. xlviij. |
| | Ext. Opium, - - - | grs. iv. |
| | Tobacco, - - . - - | grs. lxxx. |
| | Boiling Water, - - - | O.j. |
| Add | | |
| | Potassæ Nit., - - - - | grs. cxx. |
| | Potass. Arsenit, - - - | grs. cccxx. |

Take thick bibulous paper; soak it in this solution, and allow to dry. When set on fire and the flame extinguished, this paper burns slowly without flame, and emits a dense smoke which may be inhaled for the relief of asthma, often with very marked benefit. It is also useful in chronic bronchitis.—*Receipt Book of Philadelphia Hospital.*

Chloral as a Topical Application in Eczema.—A correspondent writes us that during the past year he has used, in several cases of chronic eczema, with very much satisfaction to his patients and himself, a solution of the hydrate of chloral as a topical remedy, in the proportion of one or two drachms to a pint of water, applied 2 or 3 times a day.—*Boston Med. & Surg. Jour.*

Strychnia and Phosphoric Acid in Depression.—At a recent meeting of the Ramsey Co. Medical Society, Minn., (Northwestern Medical and Surgical Journal) "Dr. C. H. Boardman, of St. Paul, read notes of a case of typho-malarial fever. The patient, a lady of 58 years, toward the close of the third week, had a pulse of 120; temp. 94 deg.; low, muttering delirium; refused food for three days; and had thirty or more involuntary passges from the bowels in the twenty-four hours. After an unsuccessful trial of various other remedies, this formidable array of grave symptoms immediately began to give place to favorable symptoms on the administration of hypodermic injections of strychnia, 1-48 gr, every three hours. Dr. C. E. Smith, reported a somewhat similar case in which the same remedy produced almost equally happy effects. Dr. H. C. Hand made favorable mention of the following prescription on account of its agreeable taste and the good results he had seen follow its use as a tonic in several cases of typhoid fever:

| | |
|---|---|
| "R.—Strychniæ, - - - - | gr. ss |
| Acidi Phosphor. Dil., - - - | fl dr. ii. |
| Syr. Limonis, - - - | fl oz. iv. |

"M. S. Teaspoonful in water every three or four hours."

We have frequently given strychnia or its equivalents and phosphoric acid in adynamic conditions with manifest advantage.—Ed. *Medical Cosmos.*

Dr. D. W. Young states that he had used quinine as a local application in the treatment of gleet, with more benefit than any other remedy. He generally used a solution of eight grains to the ounce of water.

Treatment of Phlegmon of Umbilicus.—Internally, we must combat the constipation, which is of very frequent occurrence, giving calomel in fractional doses. Externally, emollients should be used at first, but as speedily as practicable, and this plan has succeeded in two of our cases; in the first, applications of mercurial ointment with belladonna, then, as soon as possible, elastic collodion. In case of ulceration, powdered tannin and cinchona should be employed, the abdomen being covered with wadding. We have not used cauterization externally, according to the advice of M. Maynet, with a paste of chloride of zinc, nor perchloride of iron, according to Dr. Valette, of Lyons, who has employed it both internally and externally.—*Medical News and Library.*

The Air of Houses should not contain carbonic acid enough to give a precipitate when a ten-ounce bottleful is shaken with half an ounce of clear lime-water.—*Dr. R. Angus Smith.*

Case of Obstinate Vomiting.—La Tribune Medicale, March 31, 1872, contains the description of a case of obstinate vomiting in the service of Dr. A. Janot. The man was fifty-five years of age, nervo-bilious temperament, and was afflicted with persistent vomiting of all ingesta, and of serous and bilious fluids. The whole range of external and internal applications was called in aid, including ice, mustard-plasters, alcoholics, opiates, etc., but in vain. The continuance of the distressing malady almost wore him out, and the stomach seemed to be in a constant succession of contractions. Dr. Janot finally administered the following:

| | |
|---|---|
| R.—Orange flower water, - - | 80 grammes. |
| Sulphuric ether, - - - | 20 drops. |
| Castor oil, - - - - - | 30 grammes. |
| Simple syrup, - - - | 20 grammes. |

To be well shaken. Dose, a teaspoonful every hour.

After the first dose the vomiting ceased; after the fourth dose the patient took and retained a cup of broth. And from that time on, convalescence continued without any relapse. The case deserves to be remembered on account of the admirable action of the remedy.—*Half Yearly Compendium.*

Penetrating Wounds of the Knee-Joint.—A French surgeon, Dr. Champerons, according to Le Mouvement Medical, has written an elaborate memoir containing his observations on 20 cases of penetrating wounds of the knee-joint by small projectiles, treated during the late European war. His conclusions are that such injuries may be cured, and sometimes heal with astonishing facility; that in all cases, and especially when they are extensive, involving periosteum and bone, the joint must be fixed immovably as soon as possible; that explorations and drainage tubes must be employed with great caution. Twelve years ago, the late Prof. Cooper, of San Francisco, advanced views somewhat similar, and his investigations on wounds of the knee-joint have scarcely been improved on by more recent observers,—*Pacific Medical and Surgical Journal.*

Removal of Plaster of Paris Bandages.—This may be readily accomplished by wetting them with a strong solution of common salt. It causes the plaster to crumble, so that the bandage can be readily cut. It is also useful to clean the hands and nails of the operator.—*Boston Medical and Surgical Journal.*

The Posting of placards of quack medicines has been prohibited in the streets of Chicago.

Case of Diabetes Treated by Carbolic Acid.—The following case is reported by Orson Millard, M. D., in the Michigan University Medical Journal, January, 1872 :

Mr. M——, aged 65 years, called upon me about the first of February last and asked my advice in regard to a profuse discharge of urine that annoyed him very much, although it did not appear to reduce his general health in the least. The urne was clear, of a high specific gravity, and upon the application of Trommer's test revealed the fact that sugar existed in the urine. Mr. M—— assured me that some nights he was obliged to get up to micturate as often as every twenty minutes or half an hour. I at once gave him the following prescription, which had no effect whatever, so far as I could determine :

R.—Tinct. iron, - - - f oz. ij
Cod-liver oil, - - - f oz. jv. M.
Sig. take one teaspoonful before each meal.

R.—Potassii bromidi, - - - oz. j.
Aqua pura v. s, ad., - - - f oz. jv. M.
Sig. take one teaspoonful on going to bed each night.

The above medicine was taken for about ten days without the least effect upon the sugar in the urine, after which I stopped the bromide of potassium, and in its stead added two drops of carbolic acid to each dose of iron and cod-liver oil, with the happy result ot finding that the sugar began to decrease within twenty-four hours, and in ten days I found that it had entirely disappeared from the urine, and the quantity of liquid, together with the frequency of the discharge, was reduced almost to its normal standard—*Half Yearly Compendium.*

Treatment of Hypertrophied Tonsils with Chromic Acid. Dr. B. Frænkel, (Berliner Klin, Woch.) says that the application of fine needles of chromic acid to the tonsils is almost without pain or danger, and causes a notable shrinking of the parts. By frequent application of this remedy we can cause the hypertrophy to be reduced to one-half its volume. He also injects iodine into the tonsil in some cases, the iodine being dissolved in 100 parts of glycerine.—*The Doctor.*

The Oxalate of Potash in the treatment of peritonitis is recommended in the Jour. de Med. et de Chir., small does in mucilage, repeated every hour, having produced good results in three cases of puerperal origin.—*Med. Record.*

Dysentery.—Dr. N. G. Beckwith says: During a practice of twenty-two years I have used a great many different remedies for the above malady, and have never found any so perfectly reliable as the following:

R.—Beach's Neutralizing Cordial,
Fl. Ext. Witch Hazel, aa.—Mix.

Sig.—A teaspoonful every two or three hours, controlling circulation with gelseminum.

The witch hazel will also remove a film from the eye of man or beast, by making a strong decoction of the bark and applying every two hours, and is beneficial in very many things.

Phosphorus Pills.—A writer in the Druggist's Circular gives the following formula for a pill of phosphorus, by which he says they can be made of small size, at short notice, and to keep without evolving fumes: Dissolve one grain phosphorus in half a drachm of chloroform and rub in a mortar with two scruples powdered liquorice root till all the chloroform has evaporated. Add half a drachm powdered soap and work into a mass with water, and divide into twenty-four pills.—*Pacific Medical and Surgical Journal.*

Veratria in Pneumonia.—Alt treated several cases of pneu- (Centralblatt, March 30, 1872; from the Deutsch. Archiv f. Klin. Med., ix.) with veratria, which was given in doses of from one-twentieth to one-twelfth of a grain until nausea or vomiting, or until a marked effect upon the pulse and temperature, was produced. In a very few cases the local inflammation diminished after the use of the medicine, but, as a general rule, an increase in extent or in intensity of the pneumonia took place, and this was observed even when the veratria appeared to exert an influence upon the pulse and temperature.

For Offensive Breath.—The best and safest way to remove this is by using from six to ten drops of the concentrated solution of chloride of soda in a wineglassful of pure spring water, taken immediately after the operations of the morning are completed. If the odor arises from carious teeth, rinse the mouth well with a teaspoonful of the solution of the chloride in a tumbler of water. —*Journal of Health.*

How Cundurango Cures Cancer.—Mrs. Matthews, the mother of Vice President Colfax, died recently from the cancer which was cured a year ago by cundurango.—*Pacific Med. Jour.*

